# Trend and factors associated with consumption of iron-rich foods among children aged 6–23 months in Ethiopia, Evidenced from 2005–2019 Ethiopia demographic and health survey: A multivariate decomposition analysis

**DOI:** 10.1371/journal.pone.0360237

**Published:** 2026-10-16

**Authors:** Yidenek Anegagrie Birhanu, Getayeneh Antehunegn Tesema, Ejigu Gebeye Zeleke, Tilahun Yemanu Birhan

**Affiliations:** Department of Epidemiology and biostatistics, Institute of public Health, College of Medicine and Health Sciences, University of Gondar, Gondar, Ethiopia; Haramaya University, ETHIOPIA

## Abstract

**Introduction:**

Micro-nutrient deficiencies remain the major public health concern in sub-Saharan African countries including Ethiopia. In particular, adequate consumption of iron-rich foods among children aged 6–23 months in Ethiopia is very low, and is one of the reasons for the high prevalence of micronutrient deficiencies. Consumption of iron-rich food among children aged 6–23 months has changed over time in Ethiopia. However, there is scant evidence on the factors contributed for the change in the consumption of iron-rich foods.

**Objective:**

To assess the trend and factors associated with consumption of iron-rich foods among children aged 6–23 months in Ethiopia based on the 2005–2019 Ethiopian Demographic Health Surveys.

**Methods and Materials:**

A repeated cross sectional study design was employed based on the 2005, 2011, 2016 and 2019 Ethiopian Demographic Health Surveys data. A total weighted samples of 10,796 children aged 6–23 months were included. Stata version 17 statistical software was used for data management and analysis. A logit-based decomposition analysis was employed to identify factors contributing for the change in consumption of iron-rich food over time. Coefficients with the 95% Confidence Interval (CI) and P-value were reported to declare the statistical significance and strength of the contribution.

**Result:**

The consumption of iron-rich foods among children aged 6–23 months increased significantly from 10.8% (95%CI: 9.7, 11.9) in 2005 to 23.5% (95%CI:21.3, 25.6) in 2019. About 46.4% of the overall increment in the consumption of iron-rich foods among children was due to the difference in composition of women (change in endowment) across the surveys, while the difference due to the change in the effect of coefficients (change in characteristics) was insignificant. In the endowment component, the difference in composition of women with primary (23%) and secondary (10%) education, and difference in composition of women having four and above antenatal care visits (18%) were found to be significant contributing factors for the increase in the intake of iron-rich foods among young children. The increase in number of households with rich wealth status showed a 67.4% contribution for the increment of consumption of iron-rich foods during the third phase (2016–2019).

**Conclusion and recommendation:**

Consumption of iron-rich foods among children aged 6–23 months has shown a remarkable increase over time in Ethiopia. Improvement in maternal education status, increase in number of antenatal care visits and improvement in household wealth status were the main drivers for the observed change. Strengthening policies that expand women’s educational opportunities and integrate nutrition counseling into antenatal care services, and promoting household economic empowerment could further enhance iron-rich food intake. iron-rich.

## Introduction

Micronutrient deficiency which is also known as hidden hunger is one of the triple burdens of malnutrition affecting around half of under five children in the world, making it the main challenge for child nutrition [1]. These micronutrient deficiencies in children are recognized as a global public health problem, and are worse in low- and middle-income countries [2–4].

Iron deficiency is among the most common and widespread nutritional disorders worldwide, affecting many children in developing countries [5]. It affects more than 2 billion people worldwide, with children in Africa being hit hardest [6,7]**.** It has long been thought that up to half of anemia cases are due to iron deficiency [8]. According to the World Health Organization (WHO) report of 2015, almost half (47%) of under-five children were anemic, which was mainly attributed to iron deficiency [9].

In most parts of the world, children who are less than two years are the most vulnerable groups for micronutrient deficiencies, which increases the risk of death due to infectious diseases and leads to impaired physical, mental and cognitive development [10]. Infants and young children are primarily affected by iron deficiency and anemia because of the increased need for iron associated with rapid growth and brain development [11]. In addition to the increased requirement, inadequate consumption of iron-rich foods and low availability of dietary sources for iron consumption secondary to low socioeconomic status are possible reasons for iron deficiency among young children [12].

According to a report by the United Nations Children’s Fund (UNICEF) in 2019, approximately 59% of children aged 6–23 months were not fed eggs, fish, or meat, which are the most well-known sources of iron [1]. In the same report, the percentage of young children consuming these animal source foods in low and lower-middle-income countries was lower nearly by half compared to those in upper-middle-income countries [1]. In developing countries, including Ethiopia, the diets of children aged 6–23 months are often characterized by poor dietary diversity and low nutritional value foods, which are the main reasons for the decreased consumption of iron-rich foods among under two years children [13,14].

UNICEF and WHO have recommended that children aged 6–23 months should eat iron-rich foods as a daily basis, that are mainly found in animal sources, such as, eggs, meat, liver (other organs also) and fish [1]. In line with these recommendations, iron-rich, Ethiopia has been implementing programs to improve household level access to diverse, locally available nutritious foods and promoting optimal complementary feeding practices through counselling and nutrition education [15]. Despite these efforts, consumption of iron-rich foods among young children remains low, with more than half of infants not meeting the recommended intake [16], and meat consumption, which is the main source of iron worldwide, is low among African countries including Ethiopia [17].

Factors influencing the intake of iron-rich foods among young children include socioeconomic and maternal factors such as, maternal education, household wealth status, media exposure, and health service-related factors like ANC visits, place of delivery, post-natal check-ups [18–21].

Though consumption of iron-rich foods varied over time, there is a research gap at national level assessing the trend and contributing factors for the change in consumption of iron-rich foods among children aged 6–23 months. We hypothesize that improvements in parental education, parental occupation, household wealth status, media exposure, and health service-related factors like ANC visits, place of delivery, post-natal check-ups are key drivers of the observed increase in iron-rich food consumption among children aged 6–23 months. Therefore, this study aimed to explore the trends and contributing factors for the overtime change in consumption of iron-rich foods using a logit-based multivariate decomposition analysis. The findings of this study can be an input for policy makers and program planners to design a more focused and targeted intervention strategies towards those contributing factors for the change in consumption of iron-rich foods among young children.

## Methods and materials

### Data source

This study was done based on four Ethiopia Demographic and Health Survey (EDHS) data which are conducted in 2005, 2011, 2016, 2019. After submitting the purpose of the current study and receiving an approval letter from MEASURE DHS for the use of EDHS, the data was accessed from the public domain through the MEASURE DHS website (www.measuredhs.com).

### Study design and period

A repeated cross-sectional study design was employed which were conducted from April 27 to August 30 in 2005, from December 27/2010 to June 3 in 2011, from January 18 to June 27 in 2016 and from March 21 to June 28 in 2019.

### Study area

Ethiopia is found in the eastern part of Africa situated from 30 to 140 N and 330–48°E. The country has a total population of around 115 million which makes the nation the first most populous in the region and the second most populous in Africa [22]. During the time of each data collection periods Ethiopia was administratively decentralized in to nine regions and two city administrations. Each region is divided in to Zones, and each Zone again divided in to small administrative units called districts, which are further subdivided in to the lowest administrative unites, called kebeles.

In Ethiopia, animal source foods are consumed mostly only during holidays as they are considered luxurious foods instead of basic daily requirements. Moreover, animal and animal source foods that are rich in iron are used mainly for the market purpose [23].

### Source and study population

The source population of this study was all children aged 6–23 months who were in each of the year 2005, 2011, 2016 and 2019 in Ethiopia, where as children aged 6–23 months in Ethiopia who were in the selected enumeration areas of each EDHS study were considered as study population. Those children aged 6–23 months who stayed in the selected households the night before the interview were included in this study.

### Sample size determination and sampling procedure

A total weighted sample of 10,796 (2948 in 2005 EDHS, 3260 in 2011 EDHS, 3075 in 2016 EDHS and 1513 in 2019 mini-EDHS) children were included in this study.

A stratified two-stage cluster sampling technique was employed for all of the four EDHS included in this study using their respective population and housing census as a sampling frame. Each region was stratified into urban and rural areas, yielding specific number of sampling strata for each EDHS. Samples of EAs were selected independently in each stratum in two stages. In the first stage, a number of EAs were selected with probability proportional to EA size and with independent selection in each sampling stratum. The EA size is the number of residential households in the EA according to the sampling frame. A household listing operation was carried out in all of the selected EAs, and the resulting lists of households served as the sampling frame for the selection of households in the second stage.

In the second stage of selection, a fixed number of households per cluster were selected with an equal probability systematic selection from the newly created household listing. The survey interviewer interviewed only the pre-selected households. The total number of women interviewed for their children’s iron-rich food consumptions in each of EDHS were 2600 in 2005, 3173 in 2011, 2945 in 2016 and 1523 in 2019. No replacements and no changes of the pre-selected households were allowed in the implementing stages in order to prevent bias [24–27].

### Study variables

#### Dependent variable.

The dependent variable of this study was consumption of iron-rich foods measured as good consumption and poor consumption of iron-rich foods.

#### Independent variables.

**Demographic factors: –** Residency, age of child, sex of child, child is twin, birth order, age of mother.

**Socio-economic factors: –** Religion, marital status, parental education, wealth status, parental working status, media exposure, number of under five children, family size, sex of household head.

**Health service-related factors: –** Number of ANC visits, place of delivery and postnatal checkups of the child.

### Operational definition

**Good consumption of iron-rich foods**: According to the DHS guideline, if a child aged 6–23 months consumed at least one iron-rich foods item among the four animal source food items (Eggs, Meat (lamb, beef, goat, chicken, etc.), Organ meat(liver, heart or other organs), and Fish/shellfish) at any time in the last 24 h preceding the interview [28], which is applied across all the four survey rounds used in this study.

**Wealth index:** According to the DHS guideline, wealth index is classified in to quintiles: poorest, poor, middle, rich and richest. The variable was derived from the assets of each household to assess the overall living standard of the household, and the categories were computed using the Principal Component Analyses (PCA) [28].

**Media exposure:** Was created from the three variables: watching television, listening radio, and reading a newspaper, and labeled as “yes” if a woman has exposure to either of the three media sources at least once a week or “no” if a woman has exposure to none of them [28].

### Data collection and data quality controls

#### Data collection procedure.

Each of the conducted surveys was designed to provide a representative estimate based on the samples by considering the total population, geographical areas and the nine regions with the two city administrations. To collect data, each of the surveys used common type of questionnaires. These questionnaires, which are based on The DHS Program’s standard questionnaires, were adapted to reflect the population and health issues relevant to Ethiopia. From these, specific questions regarding child nutrition found within the women’s questionnaire. After the questionnaires were finalized in English, they were translated into Amharic, Tigrigna, and Afaan Oromo. Data collectors were recruited based on their language skills, academic qualifications and previous survey work experiences. The interviewers used electronic devices like tablet computers to record responses during the interviews [24–27].

#### Data quality assurance.

To assure the quality of the data, a pre-tested questionnaire and well-trained data collectors about the ways to collect data from the respondents were used. A field supervisor and additional coordinators were assigned to supervise and monitor their work and progress. The data were extracted using data extraction checklists. The variables were recoded based on different literatures, and data cleaning was done by running frequencies and cross-tabulations. Missing on the outcome and independent variables was handled according to the DHS guideline, in which “missing” and “don’t know” responses were excluded from the numerator but included in the denominator, providing a conservative estimate by avoiding overestimation [24–28].

### Data processing and analysis

#### Data processing.

Data handling procedures and extraction of relevant dependent and independent variables, consistency and completeness of Kids Record (KR) dataset was checked before the analysis. STATA version 17 software was used for data extraction, cleaning and for appending the data from 2005–2019 for trend and decomposition analysis. The data were weighted using sampling weight (v005), primary sampling unit (v021) and strata(v023) to make the it representative and to get reliable statistical estimates.

### Statistical analysis

#### Trend and decomposition analysis.

We have appended the extracted data of 2005, 2011, 2016 and 2019 EDHS data using STATA command “append using” after we kept the same variable name and labels across the surveys for the trend analysis. Trend analysis and decomposition of the change in the intake of iron-rich foods over time was conducted. The trend analysis was done separately in four phases, phase 1 (2005–2011), phase 2 (2011–2016), phase 3 (2016–2019) and the overall trend (2005–2019). We declare whether the change in consumption through time was significant or not using the 95% Confidence Interval (CI) of Proportion.

Since the outcome variable is binary, the recently developed multivariate decomposition analysis for non-linear response or logit-based multivariable decomposition analysis was employed using **mvdcmp** STATA command [29]. The analysis utilizes the output from the logistic regression model to parcel out the observed change in consumption of iron-rich foods over time into two components (due to the change in Endowment and change in Coefficient).

The main aim of multivariable decomposition was to identify factors contributing for the change in the intake of iron-rich foods among young children between 2005 and 2019. The observed change in the intake of iron-rich foods over time could be due to the composition changes or (endowments, which is explained) and/or change in effects of the characteristics (coefficients or explanatory variables), that are unexplained. Hence, the observed increase in the intake of iron-rich foods over time was additively decomposed into a characteristics (composition) component and a coefficient (or effects of characteristics) component.

#### Over all decomposition.

The proportion of differences in the consumption of iron-rich foods between any two surveys of A and B of all four consecutive EDHS can be decomposed as: –


$$\begin{array}{c} {\text{Y}}_{\text{A}}\;-\;{\text{Y}}_{\text{B}}\;=\text{ F}({\text{X}}_{\text{A}}\beta_{\text{A}})\;-\text{ F}({\text{X}}_{\text{B}}\beta_{\text{A}}) \end{array}$$

Let the 2019 EDHS be denoted as **A** and the 2005 EDHS be denoted as **B.** For logistic regression, the Logit or log-odd of consumption of iron-rich foods is taken as:


$$\begin{array}{c} \text{Logit}(\text{A})-\text{ Logit}(\text{B})=\text{F}({\text{X}}_{\text{A}}\beta_{\text{A}})\;-\text{ F}({\text{X}}_{\text{B}}\beta_{\text{B}}) \end{array}$$

$$\begin{array}{c} \begin{array}{cc} \underset{―}{\text{F}\left({\text{X}}_{\text{A}}\beta_{\text{A}}\right)-\text{ F}\left({\text{X}}_{\text{B}}\beta_{\text{A}}\right)}+ & \underset{―}{\text{F}\left({\text{X}}_{\text{A}}\beta_{\text{A}}\right)-\text{ F}\left({\text{X}}_{\text{B}}\beta_{\text{A}}\right)}\\ ↓ & ↓\\ 𝐄 & 𝐂 \end{array} \end{array}$$

$$\begin{array}{c} =\left({\text{X}}^{\text{2019}}-{\text{X}}^{\text{2005}}\right)\beta^{\text{2019}}+\left(\beta^{\text{2019}}-{\text{X}\beta }^{\text{2005}}\right){\text{X}}^{\text{2005}} \end{array}$$

The **E** component indicates to the part of the differential due to differences in endowments or characteristics. The **C** component indicates to the part of the differential due to differences in coefficients or effects of characteristics.

**Detailed decomposition: –** The equation below was applied in order to understand the unique effect of each predictor on each component of the difference. Thus, partition E(endowment) and C(coefficient) were divided in to portions, Ek and Ck (k = 1…. k) that represent the unique contribution of the k^th^ predictor on E and C, respectively.

The equation can be written as: –


$$\begin{array}{c} \text{logit}(\text{A})\;-\text{ logit}(\text{B})\;=\;(\beta_{\text{0A}}\;-\;\beta_{\text{0B}})\;+\;\Sigma {\text{X}}_{\text{ijB}}\;*\;[\beta_{\text{ijA}}\;-\;\beta_{\text{ijB}}]\;+\;\Sigma \beta_{\text{ijA}}\;*\;[{\text{X}}_{\text{ijA}}\;-\;{\text{X}}_{\text{ijB}}] \end{array}$$

Where: β_0A_ = the intercept in the regression equation for MEDHS 2019

β_0B_ = the intercept in the regression equation for EDHS 2005

β_ijA_ = the coefficient of the j^th^ category of the i^th^ determinant of MEDHS 2019

β_ijB_ = the coefficient of the j^th^ category of the i^th^ determinant of EDHS 2005

X_ijA_ = the proportion of the j^th^ category of the i^th^ determinant of MEDHS 2019

X_ijB_ = the proportion of the j^th^ category of the i^th^ determinant of MEDHS 2005

To produce accurate point estimate and standard errors, the complex sample design was considered by using the *svy* Stata command. Those factors with P-value<0.2 were entered in to multivariable decomposition analysis after binary decomposition analysis was done. From the multivariable decomposition, β coefficient with 95% confidence interval (CI) and the corresponding P-value were used to check the statistical significance of change in endowment and change in coefficient, and to identify those statistically significant factors that contribute for the change in the consumption of iron-rich foods among children aged 6–23 months. Finally, variance inflation factor (VIF) value less than 10 was used to declare as having tolerable multicollinearity.

### Ethical considerations

The study was conducted after getting ethical clearance from the University of Gondar, college of medicine and health science institutional review board (IRB). After submitting the purpose of the current study, approval letter for the use of EDHS and permission from MEASURE DHS was gained, and then the data was downloaded from the public domain through the MEASURE DHS website (www.measuredhs.com). In each of the surveys, all the original EDHS data were collected following international and national ethical guidelines. An informed consent statement was read to the parent or guardian of the child prior to participation, who may accept or decline to participate [24–27]. For details, program sees https://dhsprogram.com/methodology/Protecting-the-Privacy-of-DHS-Survey-respondents.cfm.

## Result

### Characteristics of the study population

#### Sociodemographic and economic characteristics of the study populations.

This study included a total weighted sample of 10,796 children aged 6–23 months from four survey rounds. Across all surveys, most children were aged 6–17 months. Over the study period, there was a marked improvement in maternal educational attainment, with a substantial decline in the proportion of women with no formal education and corresponding increases in primary and secondary or higher education levels. Household wealth distribution also showed modest improvement, characterized by a gradual increase in the proportion of households in the richest wealth category and a slight reduction in poorer households (Table 1).

**Table 1:** Percentage distribution of socio-demographic and economic characteristics of study populations in 2005, 2011, 2016 and 2019 Ethiopian Demographic and Health Surveys.

Characteristics	Category	Weighted frequency (%) of 2005 N = 2948	Weighted frequency (%) of 2011 N = 3260	Weighted frequency (%) of 2016 N = 3075	Weighted frequency (%) of 2019 N = 1513
Region	Tigray Afar Amhara Oromia Somalia Benishangul-Gumuz SNNPRs Gambela Harari Addis Ababa Dire Dawa	181(6.14) 28 (0.93) 678(23) 1,171(39.72) 107(3.64) 26 (0.89) 686(23.27) 8 (0.26) 7(0.23) 46 (1.56) 10 (0.35)	214 (6.58) 27 (0.83) 736 (22.57) 1,375(42.18) 89 (2.72) 39 (1.19) 677 (20.75) 11 (0.34) 9 (0.27) 73 (2.25) 11 (0.33)	229 (7.46) 30 (0.99) 565 (18.36) 1,346(43.76) 126 (4.09) 32 (1.03) 636 (20.68) 7 (0.24) 7 (0.24) 83 (2.71) 13 (0.44)	106 (7.01) 22 (1.45) 326 (21.56) 572 (37.82) 93 (6.11) 18 (1.16) 306 (20.26) 7 (0.46) 4 (0.27) 50 (3.31) 9 (0.60)
Residency	Urban Rural	228(7.74) 2,720(92.26)	451 (13.85) 2,809 (86.15)	375 (12.22) 2,700 (87.78)	429 (28.38) 1,084 (71.62)
Age of child in month	6-11 12-17 18-23	1,071(36.4) 1,115(37.8) 762(25.84)	1,197(36.71) 1,108(33.98) 955(29.3)	1,071(34.84) 1,124(36.56) 880(28.6)	485 (32.04) 561 (37.08) 467 (30.88)
Sex of child	Male Female	1,494(50.67) 1,454(49.33)	1,640(50.31) 1,620(49.69)	1,444(46.97) 1,631(53.03)	784 (51.85) 729 (48.15)
Child is twin	Yes No	53 (1.80) 2,895(98.2)	59(1.81) 3,201(8.19)	79 (2.57) 2,996(97.43)	1,484(98.10) 29 (1.90)
Birth order	First Second Third and above	514(17.43) 442(14.98) 1,992(67.59)	555 (17.01) 558 (17.13) 2,147(65.86)	609 (19.80) 506 (16.47) 1,960(63.72)	369 (24.42) 323 (21.32) 821 (54.26)
Maternal age	15-24 25-34 35-49	860(29.18) 1,430(48.51) 658(22.31)	932 (28.58) 1,672(51.3) 656 (20.13)	852 (27.70) 1,597(51.92) 626 (20.38)	479 (31.68) 761 (50.32) 272 (18.00)
Marital status	Not married Married	162(5.49) 2,786(94.51)	213 (6.53) 3,047(93.47)	158 (5.15) 2,917(94.85)	69 (4.56) 1,444(95.44)
Maternal education	No education Primary Secondary and above	2,287(77.59) 518(17.56) 143(4.85)	2,213(67.88) 908 (27.85) 139 (4.27)	1,873(60.92) 942 (30.63) 260 (8.45)	673 (44.51) 630 (41.61) 210 (13.87)
Father’s education	No education Primary Secondary and above	1,643(55.73) 969(32.87) 336(11.40)	1,640(50.24) 1,344(41.23) 276(8.53)	1,458(47.41) 1,204(39.16) 413(13.43)	
Maternal working status	Not working Working	2,061(69.93) 887(30.07)	1,580(48.49) 1,680(51.51)	1,793(58.31) 1,282(41.69)	
Father’s working status	Not working Working	47(1.60) 2,901(98.40)	70(2.24) 3,190(97.76)	500(16.25) 2,575(83.75)	
Family size	≤5 >5	1,290(43.76) 1,658(56.24)	1,540(47.25) 1,720(52.75)	1,447 (47.05) 1,628 (52.95)	829 (54.81) 684 (45.19)
Number of under five children	≤2 >2	2,508(85.08) 440(14.92)	2,733(83.84) 527(16.16)	2,607 (84.78) 468 15.22	1,354(89.49) 159 (10.51)
Wealth status	Poorest Poorer Middle Richer Richest	655(22.21) 630(21.38) 635(21.53) 558(18.93) 470(15.95)	781(23.95) 723(22.18) 64(19.86) 572(17.53) 537(16.48)	711(23.12) 644(20.95) 687(22.35) 565(18.38) 468(15.21)	305(20.17) 319(21.07) 285(18.86) 264(17.44) 340(22.47)
Media exposure	No Yes	2,554(86.62) 394(13.38)	2,500(76.71) 760(23.29)	2,477(80.55) 598(19.45)	
Sex of household head	Male Female	2,638(89.48) 310(10.52)	2,850(87.43) 410 (12.57)	2,648(86.11) 427 (13.89)	1,305(86.24) 208 (13.76)

#### Health service-related characteristics of the children and mothers of the children.

Utilization of maternal health services improved considerably between 2005 and 2019. Coverage of four or more antenatal care (ANC) visits increased steadily, with the most notable gains occurring after 2011. Facility-based delivery also increased substantially over the study period. Postnatal care utilization within two months of delivery showed an upward trend, although overall coverage remained low by 2019 (Table 2).

**Table 2:** Percentage distribution of health service-related characteristics of respondents in 2005, 2011, 2016 and 2019 Ethiopian Demographic and Health Surveys.

Characteristics	Category	Weighted frequency (%) of 2005 N = 2948	Weighted frequency (%) of 2011 N = 3260	Weighted frequency (%) of 2016 N = 3075	Weighted frequency (%) of 2019 N = 1513
Number of ANC visits	<4 ≥4	2,584(87.64) 364(12.36)	2,714(83.25) 546 (16.75)	2,057(66.88) 1,018(33.12)	849 (56.11) 664 (43.89)
Place of delivery	Home Health facility	2,760(93.64) 188(6.36)	2,888(88.59) 372 (11.41)	1,944(63.22) 1,131(36.78)	687 (45.43) 826 (54.57)
Post-natal checkup of child within 2 months	Yes No	127(4.29) 2,821(95.71)	103 (3.17) 3,157(96.83)	247 (8.02) 2,828(91.98)	198 (13.10) 1,315(86.90)

ANC: Antenatal Care.

#### Trends of iron-rich food consumption over time among children aged 6–23 months.

The trend period was divided into four phases, 2005–2011, 2011–2016, 2016–2019 and 2005–2019 to see the differences in the consumption of iron-rich foods over time and the potential source for the change in the consumption. At the national level, consumption of iron-rich foods among children aged 6–23 months increased substantially between 2005 and 2019 from 10.8% [95%CI: 9.68%, 11.92%] to 23.48% [95%CI: 21.34%, 25.62%] (a 12.7point change). The largest increase occurred between 2011 and 2016 with a 9-point change increased from 12.28% [95%CI: 11.15%, 13.41%] in 2011 to 21.27% [95%CI: 19.8%, 22.72%], while changes during 2005–2011 and 2016–2019 were relatively small and not statistically significant due to overlapping confidence intervals. Overall, the trend indicates a meaningful improvement in iron-rich food consumption over time. (Fig 1).

**Fig 1:**
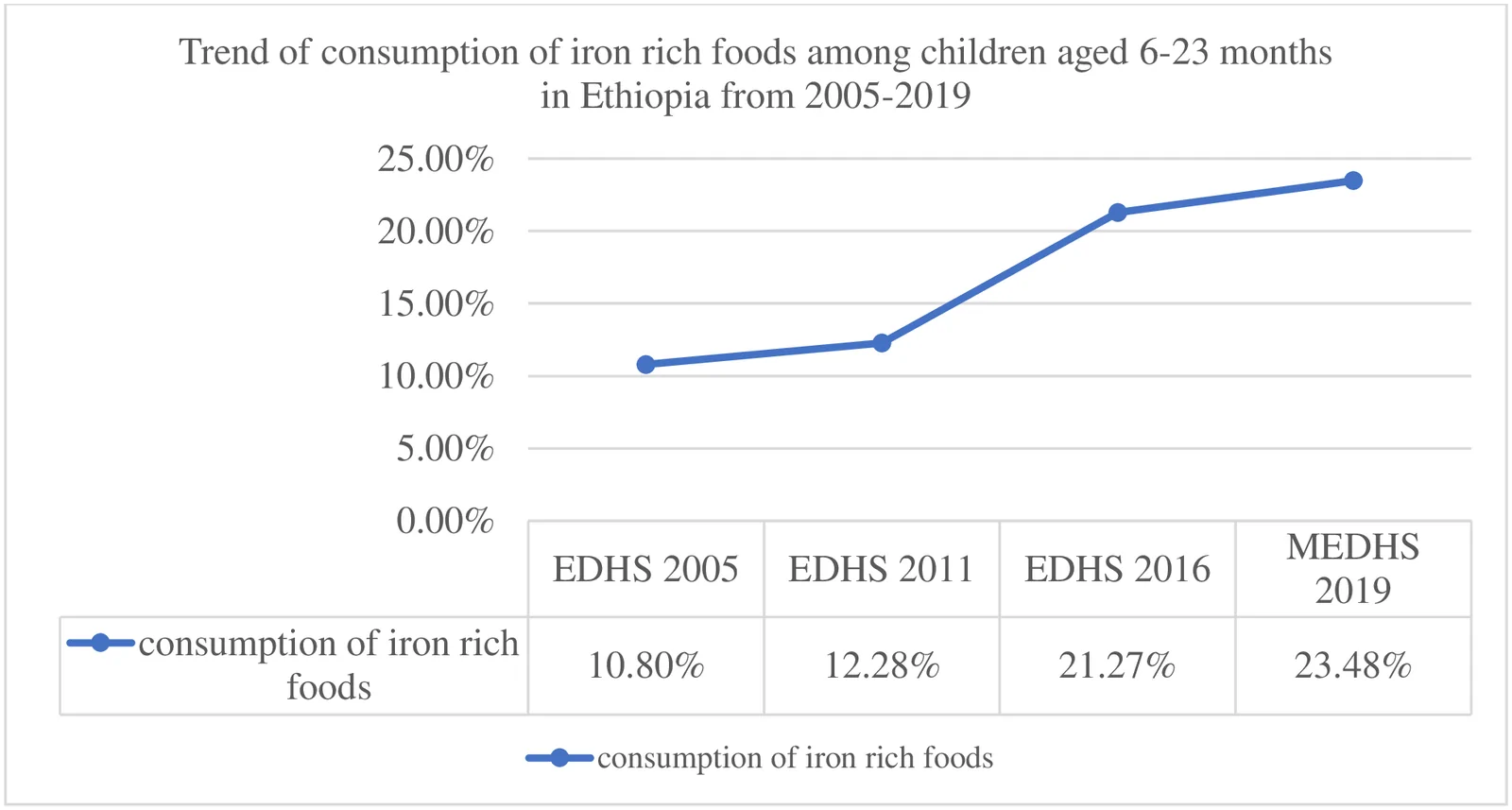
Trend of consumption of iron-rich foods among children aged 6-23 months in Ethiopia from 2005-2019.

### Trend of consumption of iron-rich foods by characteristics of the study participants

Improvements in iron-rich food consumption were observed across most sociodemographic, economic and health service–related subgroups. Higher consumption was consistently observed among children of mothers with formal education, those from wealthier households, and those whose mothers were exposed to media. Children whose mothers attended four or more ANC visits consistently showed higher consumption of iron-rich foods across surveys. These patterns underscore the importance of maternal education, household economic status, access to information, and health service utilization as key leverage points for policy and program interventions (Table 3).

**Table 3:** Trends in consumption of iron-rich foods among children aged 6-23 months by selected characteristics 2005, 2011, 2016 and 2019 Ethiopia Demographic and Health Surveys.

Characteristics	2005	2011	2016	2019	Point difference in consumption of iron-rich foods			
	N=2948	N=3260	N=3075	N=1513	2011-2005	2016-2011	2019-2016	2019-2005
Age of child								
06-Nov	6.42	10.27	16.85	19.07	3.85	6.58	2.22	12.65
Dec-17	12.6	13.09	24.28	24.69	0.49	11.19	0.41	12.09
18-23	14.33	13.85	22.82	26.61	-0.48	8.97	3.79	12.28
Residency								
Rural	9.58	11.03	18.93	20.45	1.45	7.9	1.52	10.87
Urban	25.36	20.04	38.14	31.13	-5.32	18.1	-7.01	5.77
Birth order								
First	11.26	16.49	27.23	25.99	5.23	10.74	-1.24	14.73
Second	13.9	14.67	24.67	27.42	0.77	10	2.75	13.52
Third and above	10	10.56	18.54	20.8	0.56	7.98	2.26	10.8
Maternal age								
15-24	11.67	14.76	21.3	23.62	3.09	6.54	2.32	11.95
25-34	11.5	11.74	23.34	23.72	0.24	11.6	0.38	12.22
35-49	8.13	10.09	15.98	22.57	1.96	5.89	6.59	14.44
Maternal education								
No education	8.8	9.89	17.26	13.94	1.09	7.37	-3.32	5.14
Primary	14.78	15.68	23.19	29.16	0.9	7.51	5.97	14.38
Secondary and above	28.41	27.92	43.22	37.06	-0.49	15.3	-6.16	8.65
Father’s education								
No education	8.45	11.43	17.24		2.98	5.71		
Primary	10.81	12.28	22.28		1.47	10		
Secondary and above	22.23	17.03	32.57		-5.2	15.54		
Maternal working status								
Not working	10.13	12.14	19.57		2.01	7.43		
Working	12.36	12.37	23.65		0.01	11.29		
Father’s working status								
Not working	33.88	17.03	18.37		-16.85	1.34		
Working	10.42	12.15	21.84		1.73	9.69		
Wealth status								
Poorest	7.06	8.43	15.33	11.14	1.37	6.9	-3.99	4.88
Poorer	9.44	8.97	16.85	24.95	-0.47	7.88	8.1	15.51
Middle	8.75	10.79	21.7	15.91	2.04	10.91	-5.79	7.16
Richer	11	15.41	19.31	31.69	4.41	3.9	12.38	20.69
Richest	20.35	20.78	38.14	33.17	0.43	17.36	-4.97	12.82
Media exposure								
No	9.16	10.89	18.95		1.73	8.06		
Yes	21.42	16.77	30.9		-4.65	14.13		
Family size								
≤5	11.23	14.85	23.1	27.03	3.62	8.25	3.93	15.8
>5	10.46	9.97	19.65	19.18	-0.49	9.68	-0.47	8.72
Number of under five								
≤2	11.09	12.65	22.02	24.81	1.56	9.37	2.79	13.72
>2	9.12	10.32	17.14	12.19	1.2	6.82	-4.95	3.07
Number of ANC visit								
<4	9.44	10.71	18.19	18.17	1.27	7.48	-0.02	8.73
≥4	20.43	20.03	27.51	30.28	-0.4	7.48	2.77	9.85
Place of delivery								
Home	10.2	11.4	17.37	18.91	1.2	5.91	1.54	8.71
Health facility	19.6	19.09	27.99	27.29	-0.51	8.9	-0.7	7.69
Post-natal check up								
Yes	22.58	16.86	30.63	29.29	-5.72	13.77	-1.34	6.71
No	10.27	12.12	20.46	22.61	1.85	8.34	2.15	12.34

### Trend of iron-rich food consumption by regions of Ethiopia

The consumption of iron-rich foods increased in most regions of Ethiopia over the study period; however, substantial regional variation was observed. Larger improvements were noted in urban and more developed regions, likely reflecting higher urbanization, better market access, and broader exposure to nutrition programs. Whereas minimal progress occurred in some pastoralist regions. In contrast, a declining trend was observed in the Somali region across survey years. A minimal change in Afar and a decline in Somali may reflect pastoralist mobility, limited market integration, and lower access to health and nutrition services (Fig 2).

**Fig 2:**
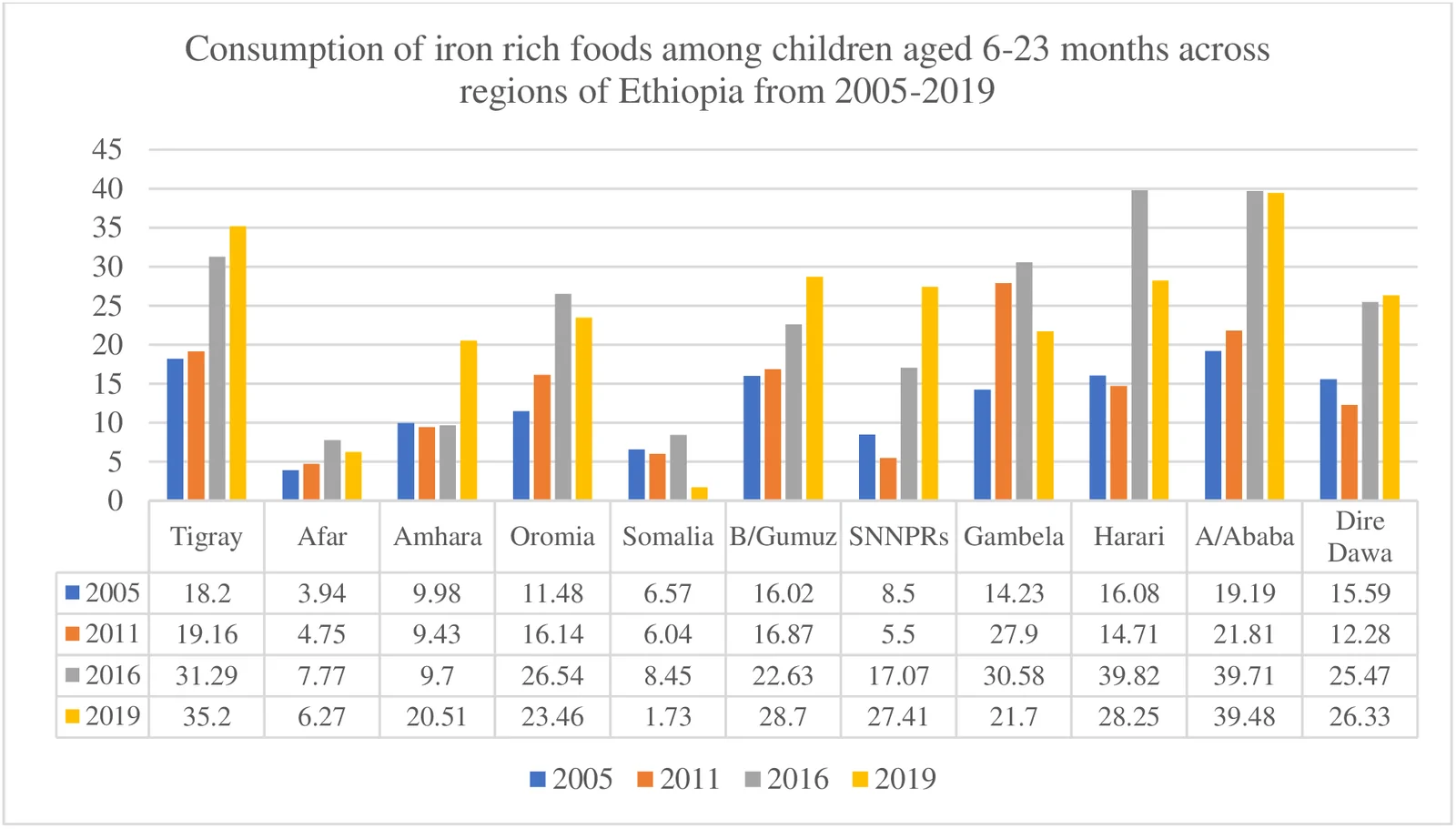
The trends in consumption of iron-rich foods among children aged 6-23 months over time across regions of Ethiopia in 2005, 2011, 2016 and 2019.

### Decomposition analysis

#### Decomposition analysis of change in the consumption of iron-rich foods among children aged 6–23 months in Ethiopia, 2005–2011.

The overall decomposition analysis showed that, both the change due to composition or characteristics and change due to the effect of explanatory variables or coefficient were insignificant, indicating that both the endowment and the coefficient have no significant effect on the increment of intake of iron-rich foods through the study period.

#### Decomposition analysis of change in the consumption of iron-rich foods among children aged 6–23 months in Ethiopia, 2011–2016.

Neither changes in population characteristics nor changes in behavioral effects significantly explained the small increase. This suggests minimal structural or behavioral shifts affecting iron-rich food consumption during this period.

#### Decomposition analysis of change in the consumption of iron-rich foods among children aged 6–23 months in Ethiopia, 2016–2019.

The significant change during this period was overwhelmingly driven by compositional shifts, in which 90.1% of the change in consumption of iron-rich foods among children aged 6–23 months was significantly explained by the change due to difference in population composition between the two survey years. Whereas, the change due to the effect of explanatory variables or coefficient was insignificant (Table 4).

**Table 4:** Overall decomposition analysis of change in consumption of iron-rich foods among children aged 6-23 months in Ethiopia, 2016-2019.

Consumption of iron-rich foods	Coefficient	P-value	95% confidence interval (CI)	Percentage (Pct.)
**E**	0.0199	0.005	[0.0062, 0.0342]	90.1 **
**C**	0.0022	0.916	[-0.0387, 0.0431]	9.9
**R**	0.0221	0.282	[-0.019, 0.0628]	

E: Endowment; C: Coefficient; R: Residual; **p-value<0.01.

In the detailed decomposition analysis, the contribution of change in the composition of richest wealth status was the only to be significant. The result revealed that about 67.39% (Coeff.: 0.015; 95%CI: 0.01, 0.024) of the change in consumption of iron-rich foods was attributed by the increase in the percentage of households with richest wealth status (Table 7). VIF value was found to be less than 10, indicating that multicollinearity between independent variables is tolerable (Table 5**).**

**Table 5:** Detailed decomposition analysis of change in consumption of iron-rich foods among children aged 6-23 months in Ethiopia, 2016-2019.

Variables	Due to difference in characteristics Coeff (95% CI)	Pct.	Due to difference in coefficient Coeff (95% CI)	Pct.
**Wealth status**				
Poorest	1		1	
Poorer	0.00012(-0.000043, 0.0003)	0.76	-0.0057(-0.211, 0.2)	-25.7
Middle	-0.00173(-0.006, 0.0024)	-7.8	0.0004(-0.014, 0.015)	1.7
Richer	-0.002(-0.003, 0.0009)	-8.6	-0.0065(-0.24, 0.23)	-29.2
Richest	0.015(0.006, 0.024) **	67.39	-0.0014(-0.0522, 0.05)	-6.31
**Family size**				
**≤5**	0.004(-0.003, 0.01)	18.14	-0.004(-0.153, 0.144)	-18.6
**>5**	1		1	
**Number of <5 children**				
**≤2**	0.0042(-0.002, 0.0101)	18.8	-0.01(-0.382, 0.362)	-46.7
**>2**	1		1	
**Place of delivery**				
Home	1		1	
Health facility	-0.001(-0.015, 0.013)	-4.24	0.004(-0.141, 0.15)	17.9
**Post-natal check up**				
Yes	0.0021(-0.003, 0.01)	9.4	0.00006(-0.003, 0.003)	0.27
No	1		1	

**Indicates p < 0.01; pct: percentage

#### Decomposition analysis of change in the consumption of iron-rich foods among children aged 6–23 months in Ethiopia, 2005–2019.

Overall, from 2005 to 2019, there has been a significant increment in consumption of iron-rich foods among children aged 6–23 months in Ethiopia. Based on the decomposition analysis result, the change in consumption of iron-rich foods was significantly explained by the change in children’s and their mothers’ characteristics (composition), whereas the change due to the difference in the effect of explanatory variables (coefficient) was insignificant (Table 6).

**Table 6:** Overall decomposition analysis of change in consumption of iron-rich foods among children aged 6-23 months in Ethiopia, 2005-2019.

Consumption of iron-rich foods	Coefficient	P-value	95% confidence interval (CI)	Percentage (Pct.)
**E**	0.06	0.003	[0.02, 0.098]	46.4**
**C**	0.068	0.098	[-0.021, 0.12]	53.6
**R**	0.127	0.076	[-0.091, 0.163]	

E: Endowment; C: Coefficient; R: Residual; **p-value<0.01.

#### Difference due to characteristics (endowment).

The decomposition analysis revealed that around 46.4% of the change in the consumption of iron-rich foods among children aged 6–23 months was attributed by the change in composition. From the compositional factors that showed a statistically significant contribution for the increment were, maternal education and number of ANC follow up.

About 23% (Coeff.:0.03; 95% CI: 0.011, 0.052) of the increase in consumption of iron-rich foods among children aged 6–23 months was attributed by the increase in percentage of women who have primary education between the two surveys. Similarly, an increase in the percentage of women with secondary or above education level, showed a significant contribution of about 10% (Coeff.: 0.013; 95%CI: 0.003, 0.023) for the change in consumption of iron-rich foods. In addition, nearly 18% (Coeff.:0.023; 95%CI: 0.001, 0.05) of the change in consumption of iron-rich food was attributed by the increase in composition of women who had four and above number of ANC visits (Table 7). Since the VIF values were less than 10, multicollinearity between independent variables was tolerable.

**Table 7:** Detailed decomposition analysis of change in consumption of iron-rich foods among children aged 6-23 months in Ethiopia, 2005-2019.

Variables	Due to difference in characteristics		Due to difference in coefficient	
	Coeff (95% CI)	Pct.	Coeff (95% CI)	Pct.
**Residency** Rural Urban	1 0.0044(−0.0161, 0.025)	3.45	1 −0.0024(−0.011, 0.0062)	−1.86
**Wealth status** Poorest Poorer Middle Richer Richest	1 −0.00044(−.0008, 0.0001) −0.001(−.0042, 0.0023) −0.003(−0.0041, 0.001) 0.01(−0.001, 0.02)	−0.35 −0.75 −1.97 6.77	1 0.016(−0.01, 0.041) 0.002(−0.0231, 0.027) 0.017(−0.01, 0.041) 0.003(−0.02, 0.025)	12.2 1.64 13.1 2.33
**Family size** ≤5 >5	0.0041(−0.003, 0.012) 1	3.55	0.0142(−0.011, 0.044) 1	11.2
**Maternal education** No education Primary Secondary and above	1 0.023(0.011, 0.048) ** 0.0131(0.003, 0.0232) *	23.12 10.32	1 0.007(−0.001, 0.0221) 0.0011(−0.005, 0.01)	5.77 0.83
**Number of ANC visit** <4 ≥4	1 0.023(0.0006, 0.045) *	17.98	1 −0.0015(−0.012, 0.01)	−1.22
**Place of delivery** Home Health facility	1 −0.018(−0.058, 0.0232)	−13.8	1 0.0014(−0.006, 0.008)	0.9

**Indicates p < 0.01; *indicates p < 0.05; pct: percentage.

## Discussion

Iron deficiency anemia (IDA) remains a major public health problem in low-and middle-income countries affecting many young children, and the burden of IDA can be alleviated by daily and adequate consumption of iron-rich foods consumption, which has a significant role in promoting children’s growth, cognitive development, school performance and future productivity [1]. Hence, this study demonstrated the trends and identified factors contributed for the change in consumption of iron-rich foods among children aged 6–23 months in Ethiopia.

The findings of this study revealed that the consumption of iron-rich foods among children aged 6–23 months has been increased over time. The main drivers contributing to this increase were improvements in household financial status, an increase in the number of ANC visits, and improvements in maternal education status. These factors collectively enhance mothers’ access to accurate nutrition information, strengthen their communication with health providers, and improve their autonomy and financial capacity to provide nutrient-rich foods. Thus, rather than acting independently, these structural determinants operate synergistically to create an enabling environment for improved child feeding practices.

In this study, the trend in the consumption of iron-rich foods among children aged 6–23 months has been grown over time, rising from 10.8% in 2005 to 23.48% in 2019. This finding was supported by other studies done in Nepal, Indonesia and Rwanda [18,19,30]. The possible reason for the overtime increment of iron-rich food consumption in Ethiopia could be because the country adopted a national nutrition strategy in 2008 and approved national nutrition programs at various points in time [31–33]. These programs were primarily concerned with ensuring adequate intake of nutrients, including micronutrients, and prioritized the nutritional needs of young children. In addition, this finding could be attributable to the establishment of health extension workers, since nutrition was one of the packages included in the program [34]. This helps to improve the nutrition of iron-rich foods among young children by delivering nutrition related advices about the different sources of iron-rich foods to the mothers of children at the health posts, household or community levels.

The overall increase in the consumption of iron-rich foods across most regions over time may indicate gradual improvements in dietary practices, possibly influenced by expanded nutrition programs and increased access to health services. The markedly larger increase observed in Addis Ababa is likely related with urban advantages, including better market access, higher socioeconomic status, and greater exposure to nutrition information. In contrast, the minimal change in Afar and the declining trend in the Somali region may be linked to persistent contextual challenges such as pastoral livelihoods, recurrent climate shocks, food insecurity, and constrained access to diverse foods and health services. These regional differences highlight persistent inequities in iron-rich food consumption and suggest that improvements have not been uniform, emphasizing the need for context-specific nutrition interventions that account for regional disparities in food systems, socioeconomic conditions, and service access.

A 46.4% change in the percentage of consumption of iron-rich foods among children aged 6–23 months over the entire study period (2005–2019) was attributed to the change in the composition of population characteristics, highlighting the importance of structural factors. An increase in composition of women with primary education revealed about 23% significant positive effect on the increment of consumption of iron-rich foods among children aged 6–23 months. Similarly, an increase in the percentage of women with secondary or higher education level showed around 10% significant positive effect on the change. The evidence from this study was supported by other studies conducted in South-Asia, Sub-Saharan, Nepal, Rwanda and Ethiopia [20,21,35–37]. This might be due to the fact that educated women are more likely to access nutrition information through media and reading materials, communicate effectively with health workers, and also higher education is often linked to higher income, enabling mothers to afford iron-rich foods for their children [36].

Approximately 18% of the increase in the consumption of iron-rich foods among children aged 6–23 months was attributed to an increase in the proportion of women who had four or more ANC visits. This finding was consistent with other studies conducted in South Asia, Nepal, and sub-Saharan, in which children whose mothers had higher number of ANC visits were more likely to consume iron-rich foods [21,36,37]. These findings may be explained by the role of ANC visits as key contact points for providing nutrition education and counseling, improving maternal knowledge of healthy child nutrition and feeding practices [38].

During the third phase of the study period (2016–2019), about 90.1% of the increase in consumption of iron-rich foods among children aged 6–23 months was due to changes in the children’s characteristics (composition), but the change in the effect of characteristics was not significant. The increase in composition of households with richest wealth status showed a 67.39% significant positive effect on the increase in consumption of iron-rich foods during this period. This finding was supported by studies conducted in South Asia, Indonesia, Sub-Saharan, Nepal, Rwanda and Ethiopia [20,21,35–37,39]. A possible explanation for this finding might be that wealthier households have greater financial capacity to purchase iron-rich animal source foods, thereby increasing children’s consumption of iron-rich foods. iron-rich

Interestingly, the decomposition analysis indicated that behavioral changes (coefficient component) were not statistically significant. This suggests that while structural factors such as maternal education, ANC attendance, and improved household wealth, their influence on actual feeding behaviors did not increase at the same pace. Thus, while progress in population characteristics supports consumption, targeted interventions are needed to accelerate behavior change and translate structural gains into consistent feeding practices.

Findings of this study have programmatic relevance. First, strategies that expand maternal education and enhance ANC-based nutrition counseling are likely to produce sustained improvements. Second, economic support for vulnerable households can reduce inequities in access to iron-rich foods. Lastly, interventions should target regions and subgroups where improvements have lagged, particularly rural or low-income communities, through community-based outreach and culturally tailored nutrition promotion.

Although this study is based on the Ethiopian context, the findings may also have relevance for other low-income countries, specifically for sub-Saharan African countries with similar health extension systems, ANC service delivery platforms, and socioeconomic transitions. In settings where maternal education levels are rising and ANC coverage is expanding, similar upward trends in iron-rich food consumption could be expected.

### Strengths and limitations of the study

Using large dataset representing the whole country Ethiopia was the strength of this study**.** However, the study considered only 24-h recall method, which might not accurately reflect participants past dietary feeding habit. Variables like media exposure, parental occupation, and father’s education were not addressed in the analysis of the last two phases (2016–2019) and (2005–2019), as these variables were not available in 2019 mini-EDHS. In addition, effect of knowledge of mothers about dietary diversity, cultural and behavioral factors were also not assessed.

## Conclusion

From 2005 to 2019, consumption of iron-rich foods among Ethiopian children aged 6–23 months increased substantially, with the greatest gains occurring between 2011 and 2016. Nearly half of this improvement was driven by changes in population characteristics, most notably maternal education, antenatal care (ANC) attendance, and household wealth, while progress in feeding behaviors lagged behind.

Based on these findings, the highest priority should be sustained investment in girls’ and women’s education, given its consistent and long-term influence on child feeding practices. Second, strengthening ANC-based nutrition counseling is critical to translate service contact into practical, actionable guidance on iron-rich complementary feeding. Third, targeted economic support for vulnerable households, such as small-livestock distribution, conditional cash transfers linked to health service use, or subsidized fortified complementary foods may help reduce financial barriers to adequate diets.

Future research should incorporate qualitative methods to explore cultural and behavioral constraints to feeding iron-rich foods and employ longitudinal dietary assessments to better capture changes in feeding practices over time. These actions are essential to sustain progress in child nutrition in Ethiopia and may offer lessons for similar settings in sub-Saharan Africa.

## Abbreviations

ANC: Antenatal Care
DHS: Demographic and Health Surveys
EAs: Enumeration Areas
EDHS: Ethiopian Demographic and Health Surveys
EPHI: Ethiopian Public Health Institute
ID: Iron Deficiency
IDA: Iron Deficiency Anemia
PCA: Principal Component Analysis
UNICEF: United Nations Children’s Fund
VIF: Variance Inflation Factor
WHO: World Health Organization
